# Construction of a prognostic model for non-small-cell lung cancer based on ferroptosis-related genes

**DOI:** 10.1042/BSR20210527

**Published:** 2021-05-27

**Authors:** Ke Han, Jukun Wang, Kun Qian, Teng Zhao, Xingsheng Liu, Yi Zhang

**Affiliations:** 1 Department of Thoracic Surgery, Xuanwu Hospital of Capital Medical University, No. 45 Changchun Street, Xicheng District, Beijing 100053, China; 2 Department of General Surgery, Xuanwu Hospital of Capital Medical University, No. 45 Changchun Street, Xicheng District, Beijing 100053, China

**Keywords:** Ferroptosis, Immune system, Non-small-cell lung cancer, Prognosis

## Abstract

We wished to construct a prognostic model based on ferroptosis-related genes and to simultaneously evaluate the performance of the prognostic model and analyze differences between high-risk and low-risk groups at all levels. The gene-expression profiles and relevant clinical data of patients with non-small-cell lung cancer (NSCLC) were downloaded from public databases. Differentially expressed genes (DEGs) were obtained by analyzing differences between cancer tissues and paracancerous tissues, and common genes between DEGs and ferroptosis-related genes were identified as candidate ferroptosis-related genes. Next, a risk-score model was constructed using univariate Cox analysis and least absolute shrinkage and selection operator (Lasso) analysis. According to the median risk score, samples were divided into high-risk and low-risk groups, and a series of bioinformatics analyses were conducted to verify the predictive ability of the model. Single-sample gene set enrichment analysis (ssGSEA) was used to investigate differences in immune status between high-risk and low-risk groups, and differences in gene mutations between the two groups were investigated. A risk-score model was constructed based on 21 ferroptosis-related genes. A Kaplan–Meier curve and receiver operating characteristic curve showed that the model had good prediction ability. Univariate and multivariate Cox analyses revealed that ferroptosis-related genes associated with the prognosis may be used as independent prognostic factors for the overall survival time of NSCLC patients. The pathways enriched with DEGs in low-risk and high-risk groups were analyzed, and the enriched pathways were correlated significantly with immunosuppressive status.

## Introduction

‘Ferroptosis’ is a type of iron-dependent programmed cell death that differs from apoptosis, cell necrosis, and autophagy. It is driven by cellular metabolism and iron-dependent lipid peroxidation. Ferroptosis is associated with ischemic organ damage and cancer. The main mechanism of ferroptosis is to catalyze the highly expressed unsaturated fatty acids on cell membranes under the action of divalent ferrous iron or lipoxygenase, which results in lipid peroxidation and induces cell death [1]. Lipid peroxides are common cellular metabolic byproducts, and phospholipid hydroperoxide glutathione peroxidase (GPX)4 is a central regulator that protects cells by neutralizing lipid peroxides. Direct inhibition of GPX4 expression or depletion of its substrates, such as glutathione and cysteine, can cause ferroptosis [2].

Lung cancer is one of the most common malignant tumor types worldwide. Morbidity and mortality from lung cancer rank first in China, and non-small-cell lung cancer (NSCLC) accounts for ∼85% of lung cancers [3]. Several reports have indicated that in NSCLC, ferroptosis can be inhibited by various mechanisms. For example, high expression of P53RRA can promote ferroptosis in lung cancer cells [4], and CDGSH iron sulfur domain 1 (CISD1) [5] and TP53 [6] have a negative regulatory effect on ferroptosis. However, whether ferroptosis-related genes are related to the prognosis of NSCLC patients is not known.

In the present study, the expression data and clinical information of NSCLC patients were downloaded from The Cancer Genome Atlas (TCGA) database and the International Cancer Genome Consortium (ICGC) database as the training set and validation set, respectively. Subsequently, differential analysis was undertaken to screen ferroptosis-related genes associated with cancer prognosis. Next, a prognostic model was constructed based on ferroptosis-related genes to predict the survival of patients. In addition, the performance of the prognostic model was evaluated and validated. Functional enrichment analysis of differentially expressed genes (DEGs) in low-risk and high-risk groups was conducted, and correlations between enriched pathways and immune status were studied.

## Materials and methods

### Data collection

The RNA sequencing (RNA-seq) data and corresponding clinical information of 1127 NSCLC patients were downloaded from the TCGA database (https://portal.gdc.cancer/) as the training set. From the ICGC database (https://dcc.icgc.org/releases/current/Projects/LUSC-US/), the data of 341 NSCLC patients were downloaded as the validation set. Simultaneously, the data of 1164 ferroptosis-related genes were downloaded from the National Center for Biotechnology Information (NCBI) (https://www.ncbi.nlm.nih.gov/) and Kyoto Encyclopedia of Genes and Genomes (KEGG) (https://www.kegg.jp/kegg/) databases.

### Construction and validation of a prognostic model based on ferroptosis-related genes

R v3.6.0 (R Institute for Statistical Computing, Vienna, Austria) was used to conduct a rank-sum test on the training set to identify DEGs between cancer tissue and paired normal tissue. The screening condition was log FC ≥1, and adjusted *P*<0.05. Subsequently, common genes between the DEGs identified and known ferroptosis-related genes were identified as candidate ferroptosis-related genes. Ferroptosis-related genes with potential independent prognostic value for the overall survival (OS) of NSCLC patients were screened by univariate Cox analysis using ‘survival’ and ‘survminer’ packages within R. Subsequently, the Search Tool for the Retrieval of Interacting Genes/Proteins (STRING) database (https://string-db.org/) was used to construct a protein–protein interaction (PPI) network for these potential ferroptosis-related genes associated with the prognosis, and correlation analysis between genes was carried out. Subsequently, the ‘glmnet’ R package was used for least absolute shrinkage and selection operator (Lasso) regression analysis to undertake dimensionality reduction to remove highly correlated genes. Then, the ferroptosis-related genes used for construction of the prognostic prediction model were screened out. The risk score of each NSCLC patient was calculated using the following formula:

$$\text{Risk score}=\beta_{1}\times {\text{Exp}}_{1}+\beta_{2}\times {\text{Exp}}_{2}+\beta_{\text{i}}\times {\text{Exp}}_{\text{i}}$$

The regression coefficient (β) was obtained through Lasso regression analysis; Exp represents the expression of each ferroptosis-related gene. The prognostic risk score model was constructed based on the expression of the ferroptosis-related genes associated with the prognosis. To evaluate the predictive ability of this model, NSCLC patients in the TCGA database were divided into ‘high-risk’ and ‘low-risk’ groups according to the median risk score; then Kaplan–Meier (KM) analysis was done to compare differences in OS between the two groups using the ‘survival’ and ‘survminer’ R packages. In addition, the ‘survivalROC’ R package was used to generate a receiver operating characteristic (ROC) curve to evaluate the prediction accuracy of the model. In addition, principal component analysis (PCA) and *t*-distributed stochastic neighbor embedding (t-SNE) analysis were undertaken using the ‘prcomp’ and ‘Rtsne’ packages of ‘stats’ in R software, respectively, to explore the distribution of patient survival in different groups. Furthermore, univariate and multivariate Cox regression analyses were used to investigate whether a risk score was an independent prognostic factor for OS in NSCLC patients.

### Analyses of functional enrichment

‘Gene ontology’ focuses on the function of genes and gene products. The ‘clusterprofiler’ R package was used to carry out this function using the Gene Ontology (GO) database. Functional-enrichment analyses on the DEGs in the training set and validation set were done using the KEGG database. Analysis of gene ontology was further divided into three domains: cellular component, molecular function, and biological process. Subsequently, the ‘GSVA’ R package was used for single-sample gene set enrichment analysis (ssGSEA) to quantify differences in scores for infiltration of immune cells and immune-related functions or pathways between high-risk and low-risk groups. Furthermore, tumor immune dysfunction and exclusion (TIDE) (http://tide.dfci.harvard.edu/login/) analysis was undertaken in the high-risk and low-risk groups to predict the immunotherapeutic effects in the two groups. Next, a single-nucleotide polymorphism (SNP) file in the training set was analyzed to investigate differences in mutations between high-risk and low-risk groups.

### Statistical analyses

Statistical analyses were carried out using R v3.6.0. KM curves and the log-rank test were used to compare survival differences between high-risk and low-risk groups. Analyses of ROC curves determined the predictive ability of the model. Then, independent prognostic factors were determined using univariate and multivariate Cox regression analyses. *P*<0.05 was considered significant.

## Results

**Figure 1:**
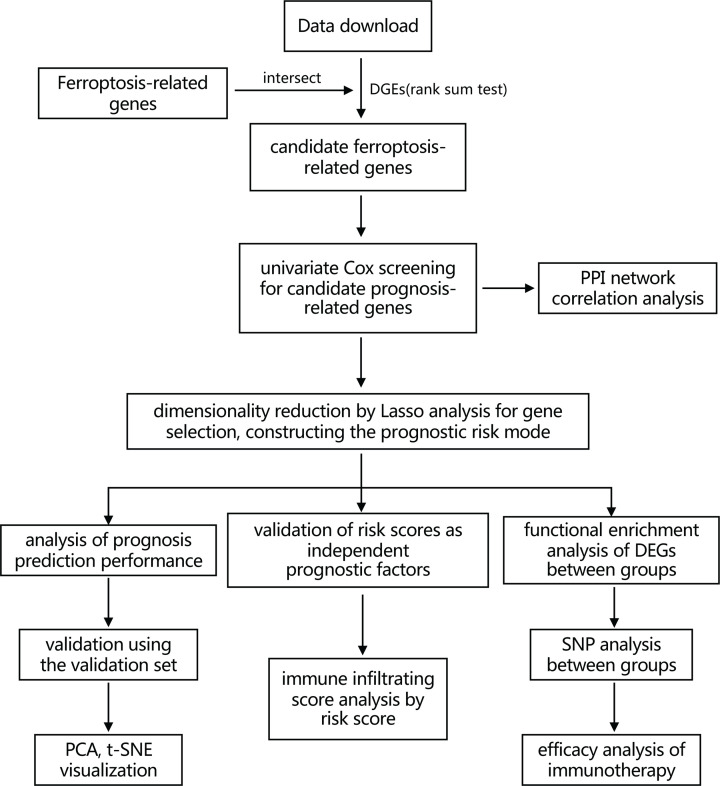
Flowchart showing the collection and analyses of data

A flowchart of the present study is shown as Figure 1. The training set comprised 1127 NSCLC patients from the TCGA database. The validation set was composed of 341 NSCLC patients from the ICGC database. The detailed clinical characteristics of these patients are summarized in Table 1.

**Table 1:** Clinical characteristics of NSCLC patients

	TCGA cohort	ICGC cohort
No. of patients	963	341
Gender		
Male	579 (60.1%)	251 (73.6%)
Female	384 (39.9%)	90 (26.4%)
Age		
≤65	NA	104 (30.5%)
>65	NA	237 (69.5%)
Pathologic-T		
T1T2	811 (84.2%)	NA
T3T4	152 (15.8%)	NA
Pathologic-N		
N0N1	834 (86.6%)	NA
N2N3	129 (13.4%)	NA
Pathologic-M		
M0	722 (75.0%)	NA
M1	241 (25.0%)	NA
Pathologic-stage		
Stage I/II	762 (79.1%)	NA
Stage III/IV	201 (20.9%)	NA

### Identification of ferroptosis-related DEGs

**Figure 2:**
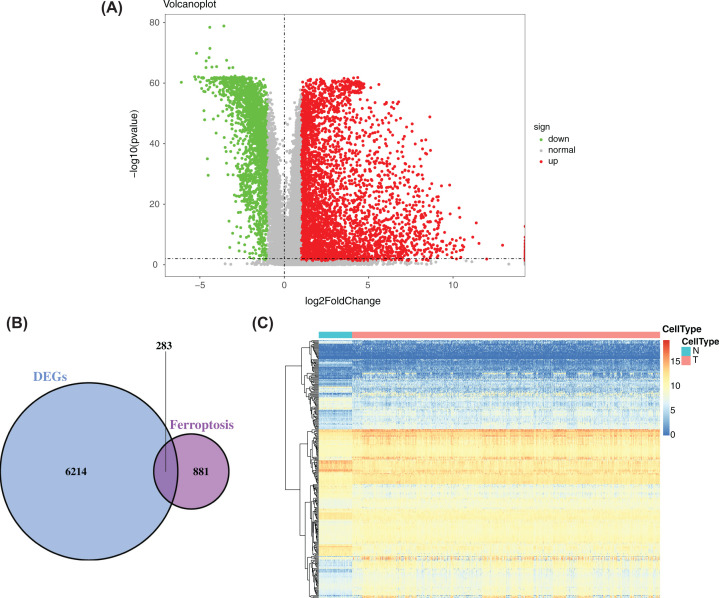
Identification of ferroptosis-related DEGs (**A**) Volcano plot of DEGs between cancer tissue and paracancerous tissue in NSCLC patients in the training set. Red represents up-regulation, blue represents down-regulation, and yellow represents intermediate transition. (**B**) Venn diagram of the intersections of DEGs and ferroptosis-related genes. (**C**) Expression distribution of 283 DEGs obtained in the training set.

The rank-sum test was used to compare DEGs between cancer tissue and paracancerous tissue in the training set and resulted in 6497 DEGs. Volcano plots were drawn using the ‘ggplot2’ R package (Figure 2A). A total of 1164 ferroptosis-related genes were obtained from NCBI and KEGG databases and were intersected with DEGS obtained previously. Finally, 283 candidate ferroptosis-related genes were obtained (Figure 2B), and they were used in a subsequent factor analysis. Subsequently, we made a heatmap of the characteristics of 283 candidate ferroptosis-related genes that showed the differential expression of ferroptosis-related genes between cancer tissues and adjacent tissues (Figure 2C).

### Identification of ferroptosis-related genes associated with NSCLC prognosis using univariate cox regression analysis

**Figure 3:**
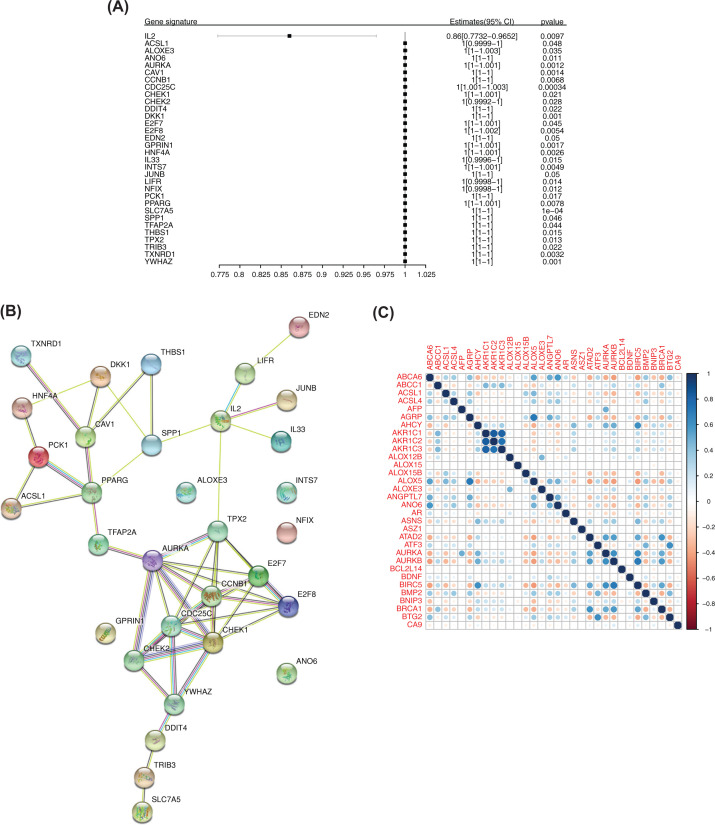
Ferroptosis-related genes associated with the prognosis (**A**) Forest map of the results of univariate Cox analysis of 32 genes. (**B**) PPI network of 32 genes. (**C**) Correlation analysis of 32 genes. Blue represents positive correlation, and red represents negative correlation.

A total of 283 candidate ferroptosis-related genes were screened by univariate Cox analysis, and 32 potential ferroptosis-related genes significantly associated with the prognosis of patients were obtained (Figure 3A). The first four genes were selected according to significance, and survival curves were constructed based on patient survival (Supplementary Figure S1).

### Construction of a PPI network based on ferroptosis-related genes associated with the prognosis and correlation analysis

A PPI network of 32 genes was constructed using the STRING database. Protein interaction was strong and many kinds of interactions were noted (Figure 3B). Correlation analysis between genes in the training set was done according to gene expression (Figure 3C).

### Establishment of a prognostic risk model of NSCLC based on ferroptosis-related genes

**Figure 4:**
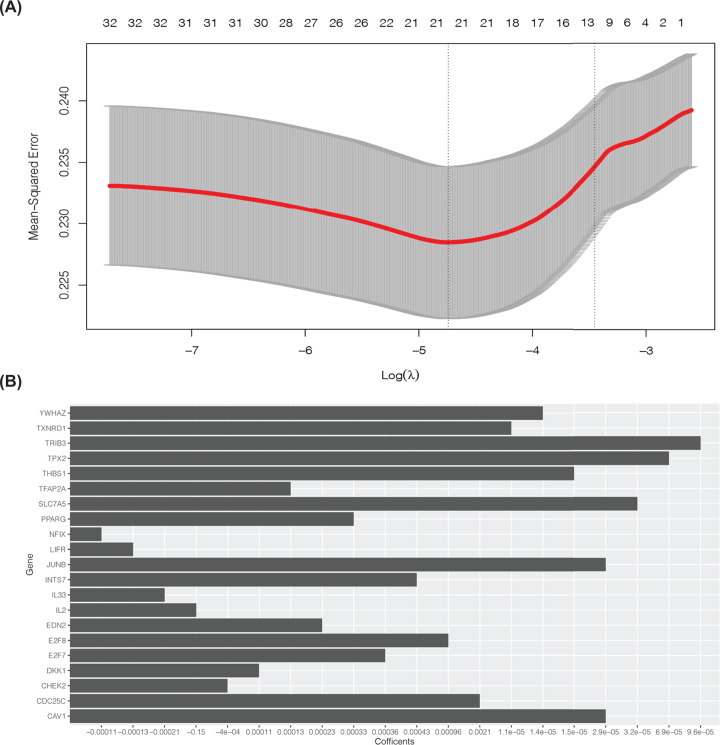
Dimensionality reduction by Lasso analysis for gene selection (**A**) Lasso analysis of 32 genes. (**B**) Twenty-one ferroptosis-related genes associated with the prognosis were obtained for construction of a risk-prediction model.

To reduce the high correlation between ferroptosis-related genes, Lasso regression analysis was undertaken for dimensionality reduction on 32 genes (Figure 4A). Thus, 21 related genes were retained for construction of the prognostic risk model (Figure 4B).

### Performance evaluation of the prognostic risk model based on ferroptosis-related genes in the training set

**Figure 5:**
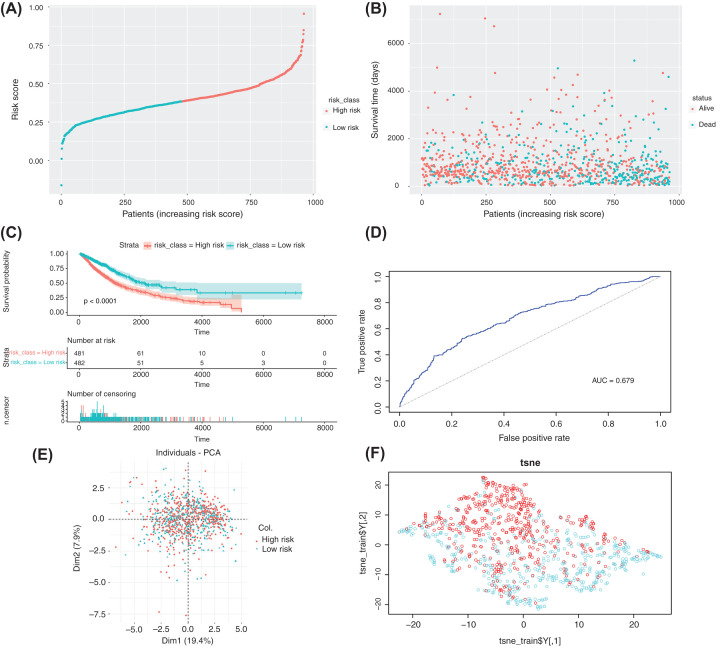
Construction of a risk-prediction model of the training set and analyses of model performance (**A** and **B**) Risk chart of the training set. Distributions of risk scores and survival times of samples from the TCGA database. (**C**) A KM curve was used to analyze differences in survival time between high-risk and low-risk groups, which were divided according to the risk model constructed by the training set, *P*<0.0001. (**D–F**) ROC curves, PCA, and t-SNE visualization demonstrating the performance of the risk model constructed using the training set.

First, according to the median risk score, the samples in the training set were divided into the high-risk group (*n*=481 cases) and low-risk group (*n*=482 cases) (Figure 5A). A risk chart showed that patients in the high-risk group had shorter survival times (Figure 5B). Analyses of KM curves showed that the survival time in the low-risk group was significantly longer than that in the high-risk group (Figure 5C). In addition, analyses of ROC curves (AUC) = 0.679 (Figure 5D), PCA (Figure 5E), and t-SNE (Figure 5F) showed that the model had good prediction ability, and it could distinguish between high-risk and low-risk groups.

### Performance evaluation of the prognostic risk model based on ferroptosis-related genes in the validation set

**Figure 6:**
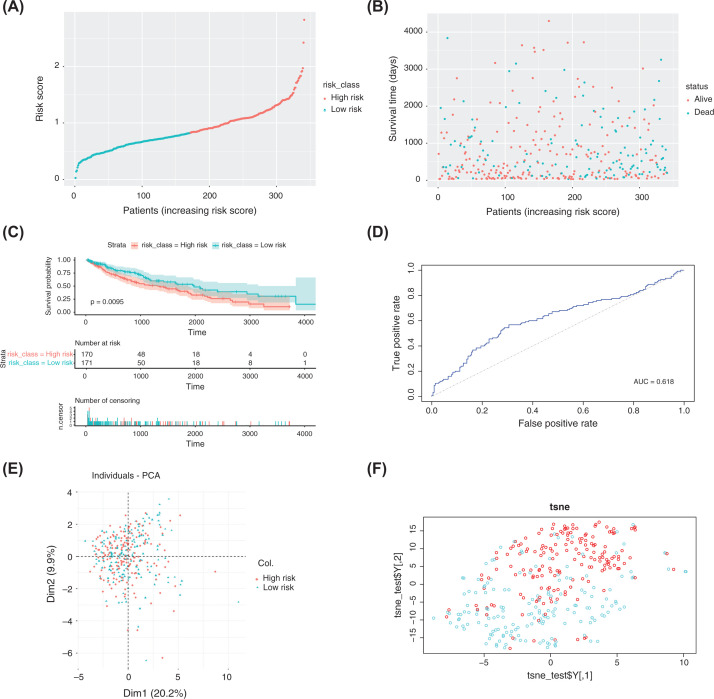
Construction of a risk-prediction model of the validation set and analyses of model performance (**A** and **B**) Risk chart of the validation set. Distributions of risk scores and survival times of samples from the ICGC database. (**C**) A KM curve was used to analyze differences in survival time between high-risk and low-risk groups, which were divided according to the risk model constructed using the validation set, *P*<0.0095. (**D–F**) ROC curves, PCA, and t-SNE visualization demonstrating the performance of the risk model constructed using the validation set.

To test the predictive ability of the model, the training set was used to evaluate the risk-prediction model. First, the median risk score was calculated based on the formula of the risk score established using the training set, and then the samples in the validation set were divided into the high-risk group (*n*=170 cases) and low-risk group (*n*=171 cases) (Figure 6A). The results were identical to those of the training set. The risk chart indicated that the patients in the high-risk group had a worse prognosis (Figure 6B). The KM curve showed that the survival time in the low-risk group was significantly longer than that in the high-risk group (Figure 6C). In addition, analyses of ROC curves (AUC = 0.618) (Figure 6D), PCA (Figure 6E), and t-SNE (Figure 6F) showed that the model had good prediction ability and could distinguish between high-risk and low-risk groups.

### Determination of the risk score as an independent prognostic factor for NSCLC

**Figure 7:**
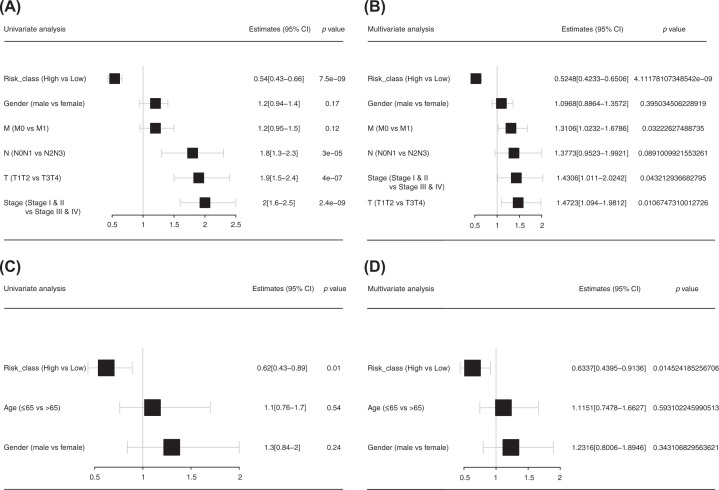
Risk score as an independent prognostic factor (**A** and **B**) Univariate and multivariate Cox analyses of the training set. (**C** and **D**) Univariate and multivariate Cox analyses of the validation set.

Univariate and multivariate Cox analyses were undertaken to ascertain if the risk score was an independent prognostic factor relative to other variables in the training set and validation set. In the univariate Cox analysis of the training set (Figure 7A), the hazard ratio (HR) was 0.54 (95% confidence interval (CI): 0.43–0.66, *P*=7.5 × 10^−9^), and the tumor–node–metastasis (TNM) stages were also prognostic factors for NSCLC patients. In the multivariate Cox analysis (Figure 7B), the risk score remained a significant independent prognostic factor, the HR was 0.52 (95%CI: 0.42–0.65, *P*=4.11 × 10^−9^), and the TNM stages were also prognostic factors for NSCLC patients. In the validation set, in the univariate Cox analysis (Figure 7C), the HR was 0.62 (95%CI: 0.43–0.89, *P*=0.01), and in the multivariate Cox analysis (Figure 7D), the HR was 0.63 (95%CI: 0.44–0.91, *P*=0.01). Therefore, compared with other traditional prognostic factors for NSCLC patients, the risk score could be used as an independent prognostic factor for NSCLC patients.

### Functional-enrichment analysis of DEGs between high-risk and low-risk groups

**Figure 8:**
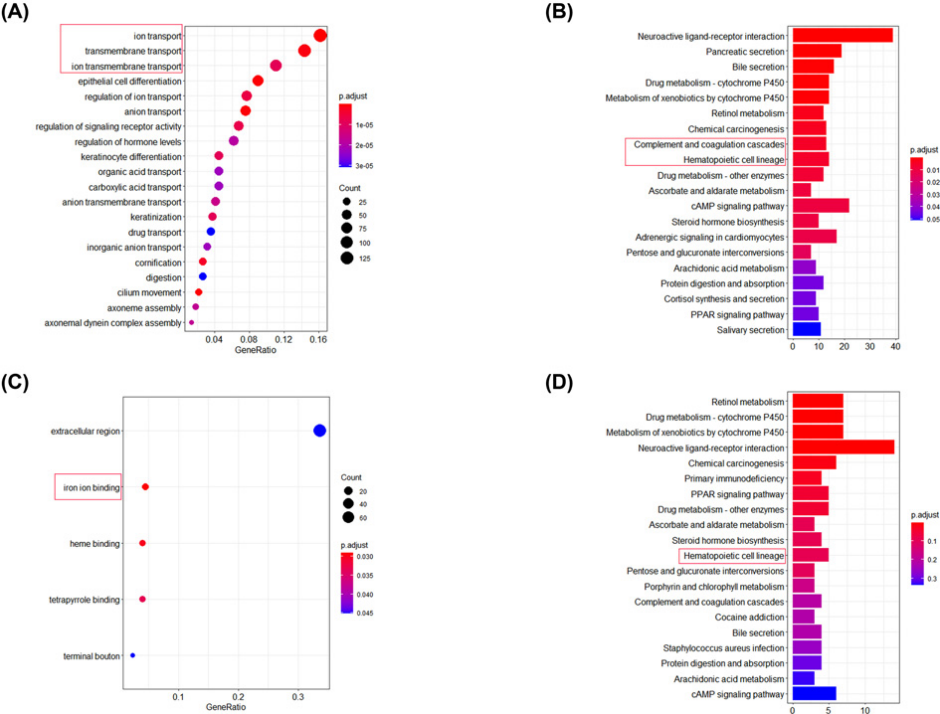
Functional-enrichment analysis of DEGs (**A** and **B**) Functional-enrichment analyses of the training set using the GO and KEGG databases. (**C** and **D**) Functional-enrichment analyses of the validation set using the GO and KEGG databases.

Functional-enrichment analysis of DEGs between high-risk and low-risk groups was undertaken in the training set and validation set. Analyses of functional enrichment using the GO and KEGG databases were undertaken to explore differences in biological functions, and a false discovery rate (FDR) <0.05 was the significance threshold. In enrichment analysis using the GO database (Figure 8A,C), DEGs were significantly enriched in BPs associated with iron ions in the training set (‘ion transport’, ‘transmembrane transport’, and ‘ion transmembrane transport’) and in the validation set (‘iron ion binding’). In enrichment analysis using the KEGG database (Figure 8B,D), DEGs were enriched in immune-related pathways in the training set (‘complement and coagulation cascades’ and ‘hematopoietic cell lineage’) and in the validation set (‘hematopoietic cell lineage’).

### Correlation analysis for infiltration of immune cells

**Figure 9:**
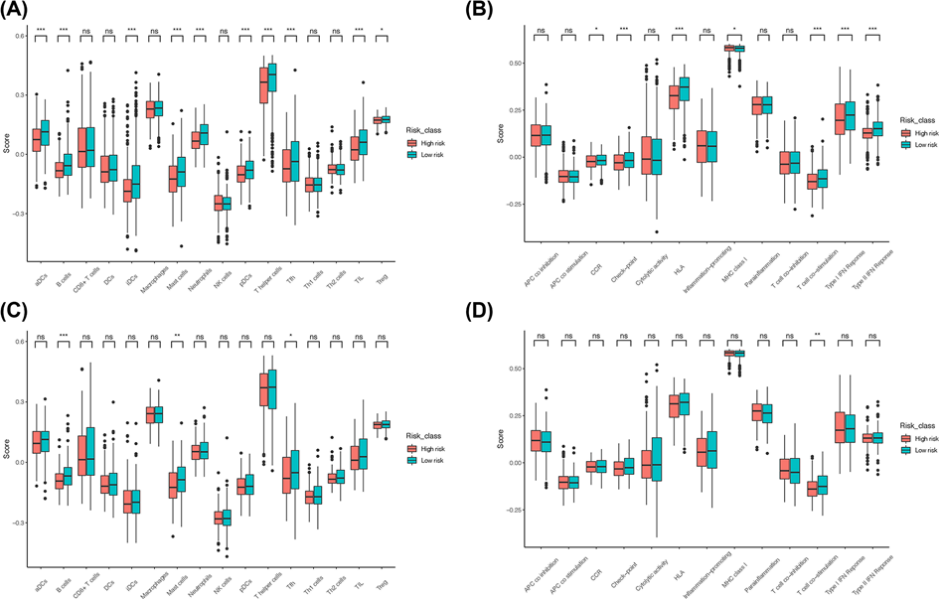
Analysis of ferroptosis-related genes associated with immune-related functions in high-risk and low-risk groups (**A** and **B**) Analysis of differences in immune cells and immune-related pathways between the high-risk and low-risk groups in the training set. (**C** and **D**) Analysis of differences in immune cells and immune-related pathways between the high-risk and low-risk groups in the validation set.

We wished to study further the correlation between the risk score and immune status. ssGSEA was used to quantify scores for immune cells and immune-related pathways. Activated dendritic cells (aDCs), B cells, immature dendritic cells (iDCs), mast cells, neutrophils, plasmacytoid dendritic cells (pDCs), T helper (Th) cells, T follicular helper (Tfh) cells, tumor-infiltrating lymphocytes (TILs), T regulatory cells (T_regs_), C-C chemokine receptors (CCRs), checkpoints, human leukocyte antigen (HLA), major histocompatibility complex (MHC) class I, T-cell costimulation, the type-I interferon (IFN) response, and type-II IFN response (Figure 9A,B) were significantly different between the two groups in the training set, whereas B cells, mast cells, Tfh cells, and T-cell costimulation were significantly different between the two groups in the validation set (Figure 9C,D).

### Prediction of immunotherapy efficacy

**Figure 10:**
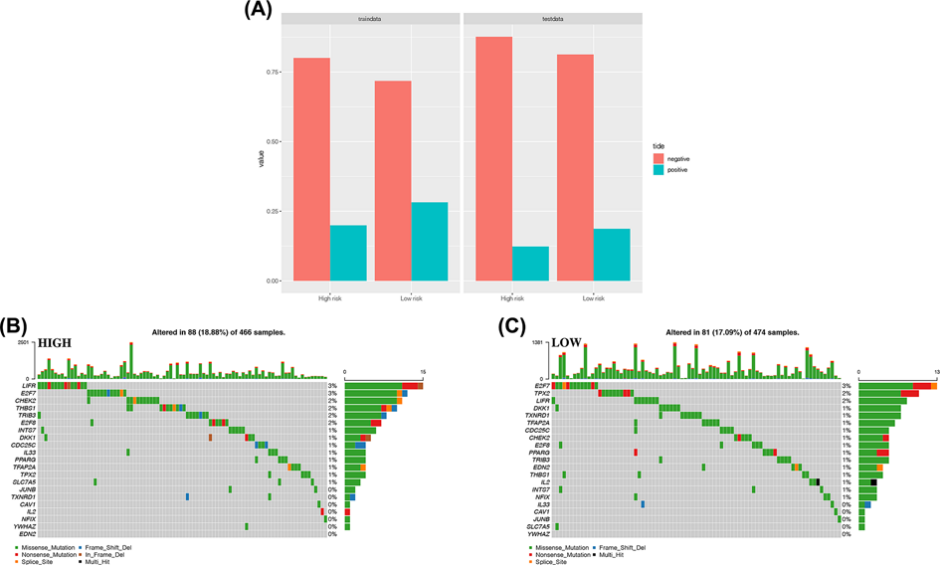
Predictive analysis of immunotherapy and analysis of mutations of ferroptosis-related genes associated with the prognosis (**A**) Proportions of samples with effective immune therapy in the high-risk samples and low-risk samples in the TIDE prediction results. (**B**) Mutations in 21 ferroptosis-related genes in high-risk samples. (**C**) Mutations in 21 ferroptosis-related genes in low-risk samples.

TIDE prediction was carried out on the two groups in the training set and validation set. Results showed that immunotherapy might have higher efficacy in the low-risk group than in the high-risk group (Figure 10A).

### Mutation analysis of ferroptosis-related genes associated with the prognosis

The SNP file of the training set was used to visualize mutations of the 21 ferroptosis-related genes in high-risk and low-risk groups. The frequency of gene mutation in these two groups was not high, and significant differences were not found between the two groups. Therefore, the effect of the 21 ferroptosis-related genes on the sample risk score was not strongly correlated with gene mutation (Figure 10B,C).

## Discussion

As a recently identified iron-dependent programmed process for cell death, ferroptosis has a unique mechanism of occurrence and resistance, and its importance in tumor therapy has been demonstrated. An increasing number of therapies targeting ferroptosis are under study, and the inhibition of ferroptosis in tumor cells has also attracted widespread attention, but specific studies investigating the mechanism are lacking. We focused on correlations between ferroptosis-related genes and the prognosis of NSCLC patients.

We systematically studied expression of 1164 ferroptosis-related genes in tumor tissues in people with NSCLC and their relationships with the OS of patients. We used univariate Cox and Lasso regression analyses to identify 21 ferroptosis-related genes associated with the prognosis and constructed a prediction model. Based on the median risk score, we employed KM curves, ROC curves, a risk-score chart, and heatmaps to evaluate the predictive ability of this model. Univariate and multivariate Cox regression analyses were also undertaken to determine if the risk score calculated by the prognostic model was an independent prognostic factor for the OS of NSCLC patients. In addition, differences in gene-set enrichment, immune-cell infiltration, and somatic mutations between the high-risk and low-risk groups were studied.

Multiple genes used in the present study to construct the prognostic model were closely related to the occurrence and development of NSCLC. Specifically, overexpression of caveolin 1 (CAV1) can activate the signal transducer and activator of transcription-3 (STAT3) pathway to induce the growth, proliferation, migration, and epithelial–mesenchymal transition (EMT) of NSCLC cells [7]. Cell division cycle 25C (CDC25C) can inhibit apoptosis in various tumor types, including NSCLC, through regulation of the G2/M-phase transition and FAS pathway [8]. Studies have shown that checkpoint kinase 2 (CHEK2) has a critical role in repairing DNA damage, and that CHEK2 overexpression is closely related to the occurrence of lung cancer [9]. Dickkopf1 (DKK1) protein regulates the proliferation and invasion of cells. In *in vitro* experiments, up-regulation of DKK1 expression in the SBC-3 lung cancer cell line enhanced the proliferation, migration, and invasion of cells, and colony formation; in *in vivo* experiments, up-regulation of DKK1 expression promoted the metastasis of lung cancer cells to bone [10]. Studies have shown that inhibition of E2F transcription factor 7 (E2F7) expression can inhibit the viability of lung cancer cells, inhibit the formation, migration, and invasion of tumor colonies, and promote tumor-cell apoptosis [11]. E2F transcription factor 8 (E2F8) is a key transcription factor involved in several biological processes and is a direct target gene of microRNA (miR)-223-5p. Overexpression of miR-223-5p can significantly reduce expression of the messenger RNA (mRNA) and protein of E2F8 in NSCLC cells, thus inhibiting the proliferation, migration, and invasion of lung cancer cells [12]. Interleukin (IL)-33 is a member of the IL-1 superfamily and is produced by Th2-related cytokines. IL-33 up-regulates glucose transporter 1 (GLUT1) expression in NSCLC through activation of the ST2 pathway, thereby enhancing glucose uptake and glycolysis and promoting the growth and metastasis of lung cancer cells [13]. Studies have shown that nuclear factor IX (NFIX) plays a major regulatory part in multiple genes involved in migration and invasion pathways. Silencing of NFIX expression can inhibit the proliferation, migration, and invasion of lung cancer cell lines [14]. Studies have shown that activation of peroxisome proliferator-activated receptor-gamma (PPARGγ) may inhibit the progression of lung squamous cell carcinoma (LSCC) through the regulation of upstream and downstream marker genes of LSCC, and these genes are involved in tumor-cell proliferation and protein polyubiquitination/ubiquitination [15]. Inositol 1,4,5-trisphosphate 3-kinase (ITPKA), a member of the inositol polyphosphate kinase (IPK) family, regulates the level of inositol polyphosphate, which is critical for transduction of cell signals. Studies have shown that transcription factor AP-2 alpha (TFAP2α) promotes the proliferation, invasion, and metastasis of lung adenocarcinoma cells through ITPKA overexpression, which is induced by an interaction with Drebrin 1 (associated with cancer metastasis) and activation of EMT [16]. Thrombospondin 1 (THBS1) has antiangiogenic effects. Studies have shown that THBS1 has a tumor-suppressive role in lung adenocarcinoma, and THBS1 is usually insufficiently expressed in lung cancer tissues [17]. Targeting protein for Xklp2 (TPX2) is positively correlated with the metastasis and growth of tumor cells as well as the clinical stage in NSCLC. Overexpression of TPX2 indicates a poor prognosis for patients. *In vitro* experiments have shown that up-regulation of TPX2 expression activates EMT and promotes the expression and activity of matrix metalloproteinase-2 (MMP2) and MMP9, thereby increasing the migration and invasiveness of NSCLC cells [18]. Studies have demonstrated that the interaction between tribbles homolog 3 (TRIB3) and β-catenin increases the recruitment of β-catenin to the promoter region of Wnt regulatory genes, thereby promoting the migration, invasion, and EMT of lung cancer cells [19]. Studies have shown that using small interfering RNAs to knock down thioredoxin reductase 1 (TXNRD1) expression increases the basal level of reactive oxygen species and increases the sensitivity of lung cancer cells to radiotherapy [20]. In NSCLC tissues, the transcription and expression of tyrosine 3-monooxygenase/tryptophan 5-monooxygenase activation protein zeta (YWHAZ) are increased significantly. YWHAZ overexpression is associated with clinical stage as well as lymph-node and distant metastasis in NSCLC patients. *In vitro* experiments have shown that silencing of YWHAZ expression can inhibit the migration and invasion of NSCLC cells [21].

Although ferroptosis in tumor cells has been a ‘hot topic’ in recent years, few studies on the potential regulatory mechanism between tumor immunity and ferroptosis are available. Therefore, we undertook functional-enrichment analyses using GO and KEGG databases on DEGs between two risk groups. We showed that immune-related pathways were enriched. Next, quantification (scoring) of immune-cell infiltration and immune-related pathways or functions using ssGSEA showed that the levels of mast cells and Tfh cells in the high-risk group in the training set and validation set were low, and that T-cell costimulatory pathways were inhibited. Studies have shown that mast cells exhibit weak phagocytosis and can cause lysis and destruction of tumor cells upon contact with lung cancer cells [22], and that Tfh cells (as a T-cell subset) participate in antitumor immunity [23]. In addition, activation of T-cell costimulatory pathways plays an important part in the activation of antitumor cluster of differentiation (CD)4+ T cells and CD8+ T cells [24]. We hypothesized that this result may be due to the release of different signals by cells that had undergone ferroptosis, such as lipid mediators, which inhibit mast cells, Tfh cells, and related immune pathways/functions, thereby promoting tumor progression. This result is consistent with the TIDE prediction, and the efficacy of immunotherapy may have been poor in the high-risk group. In addition, based on the SNP file, mutations of ferroptosis-related genes associated with the prognosis in the high-risk and low-risk groups were analyzed: no significant differences in gene mutations between the two groups were documented. Therefore, the effect of these 21 genes on the risk score was not strongly correlated with gene mutation. For example, *YWHAZ* had only one mutation in all samples, and its regulatory effect on the risk score was therefore believed to not be correlated with gene mutation, which excludes one direction of analysis for further in-depth studies. In addition, other possible factors warranting investigation include choroidal neovascularization (CNV) factor and binding of target genes influencing RNA and proteases.

## Conclusions

A risk-prediction model was constructed using ferroptosis-related genes. This model had relatively good predictive ability in the training set and validation set and could facilitate traditional TNM staging in prognosis prediction. In addition, the risk score based on ferroptosis-related genes could better differentiate the immune status of patients and predict differences in immunotherapeutic efficacy, thereby providing a new concept for immunotherapy in NSCLC patients. This result suggests that the correlation between ferroptosis-related genes and NSCLC prognosis may involve tumor immunity, which requires further study. We believe that, with exploration of the mechanism of ferroptosis in tumor cells, targeted ferroptosis in tumor cells may become a treatment option for NSCLC patients.

## Supplementary Material

Supplementary Figure S1

## Data Availability

The datasets used and/or analyzed during the current study are available from the corresponding author upon reasonable request.

## Abbreviations

CAV1: caveolin 1
CNV: choroidal neovascularization
DEG: differentially expressed gene
GLUT1: glucose transporter 1
Lasso: least absolute shrinkage and selection operator
MMP-2: matrix metalloproteinase-2
NFIX: nuclear factor IX
NSCLC: non-small-cell lung cancer
PPARGγ: peroxisome proliferator-activated receptor-gamma
ssGSEA: single-sample gene set enrichment analysis
STAT3: signal transducer and activator of transcription-3
