# Supplementary material for: Construction of a prognostic model for non-small-cell lung cancer based on ferroptosis-related genes
Source: Biosci Rep. 2021 May 27;41(5):BSR20210527. doi: 10.1042/BSR20210527 (PMC8170652; doi:10.1042/BSR20210527)
Supplement: Supplementary Figure S1 [file BSR-2021-0527_supp.pdf]

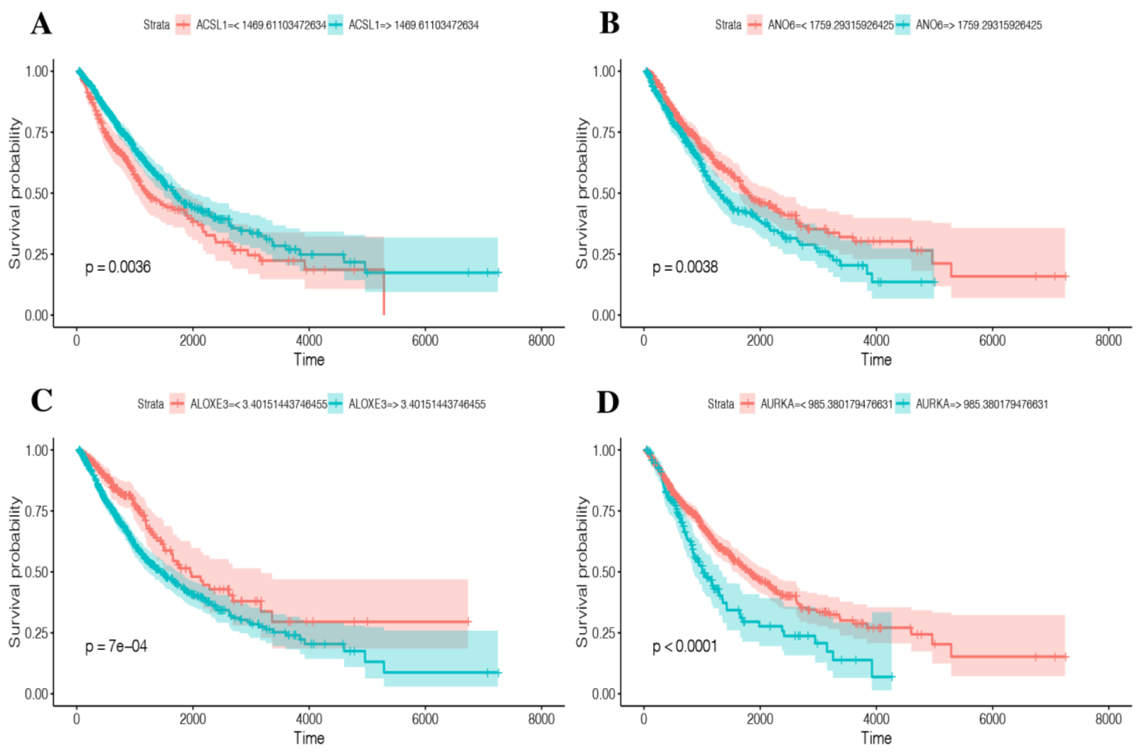

Supplementary Fig.1 Survival curves about four DEGs selected from 32 candidate ferroptosis-related genes.

（A）Survival curve of ACSL1 show that NSCLC patients with high expression had better survival than those with low expression, p=0.0036. (B) Survival curve of ANO6 show that NSCLC patients with low expression had better survival than those with high expression, p=0.0038. (C) Survival curve of ALOXE3 show that NSCLC patients with low expression had better survival than those with high expression, p=7e-04. (D) Survival curve of AURKA show that NSCLC patients with low expression had better survival than those with high expression, p ＜0.0001.
